# Medical Devices for Tremor Suppression: Current Status and Future Directions

**DOI:** 10.3390/bios11040099

**Published:** 2021-03-30

**Authors:** Jiancheng Mo, Ronny Priefer

**Affiliations:** Massachusetts College of Pharmacy and Health Sciences University, School of Pharmacy, Boston, MA 02115, USA; jmo1@stu.mcphs.edu

**Keywords:** tremor, medical devices, transcutaneous electrical nerve stimulation, electrical stimulation systems, wearable orthoses, assistive feeding devices

## Abstract

Tremors are the most prevalent movement disorder that interferes with the patient’s daily living, and physical activities, ultimately leading to a reduced quality of life. Due to the pathophysiology of tremor, developing effective pharmacotherapies, which are only suboptimal in the management of tremor, has many challenges. Thus, a range of therapies are necessary in managing this progressive, aging-associated disorder. Surgical interventions such as deep brain stimulation are able to provide durable tremor control. However, due to high costs, patient and practitioner preference, and perceived high risks, their utilization is minimized. Medical devices are placed in a unique position to bridge this gap between lifestyle interventions, pharmacotherapies, and surgical treatments to provide safe and effective tremor suppression. Herein, we review the mechanisms of action, safety and efficacy profiles, and clinical applications of different medical devices that are currently available or have been previously investigated for tremor suppression. These devices are primarily noninvasive, which can be a beneficial addition to the patient’s existing pharmacotherapy and/or lifestyle intervention.

## 1. Introduction

Tremors, as defined by the task force of the International Parkinson and Movement Disorder Society (IPMDS), are an involuntary, rhythmic, oscillatory movement of a body part [1]. Essential tremor (ET) is recognized as the most prevalent pathological tremor among adults, affecting about 0.9% of the global population [2]. However, the true prevalence of ET may be higher, as it is believed that these patients may not seek medical attention [3]. Tremors, usually asymmetrically distributed, are frequently seen in patients with Parkinson’s disease (PD), which affects more than six million individuals worldwide [4]. The presence of resting tremor supports the diagnosis of PD [5]. Different clinical subtypes and classifications of tremor disorders have also been identified [1]. The etiologies of tremor include other neurodegenerative diseases such as Wilson’s disease, chromosomal aneuploidy, mitochondrial genetic disorders, infectious and inflammatory diseases, endocrine and metabolic disorders, neuropathies and spinal muscular atrophies, toxin-/drug-induced tremor pathology, and brain neoplasms and injury, as well as several environmental causes [1].

Tremors impact many aspects of the patient’s daily living and interfere with many physical activities at home and in the workplace [6,7,8,9,10]. One clinical-epidemiological study compared the quality of life, including physical and psychosocial aspects, between patients with ET and PD using the Quality of Life in Essential Tremor (QUEST) questionnaire [11]. Patients with ET had a higher QUEST total score and QUEST physical subscore than patients with PD (*p* < 0.05). This suggests that patients with ET suffers significantly more physical and psychosocial impairment than those with PD [11]. Additionally, among patients suffering from tremor, their psychological strain may be significantly more affected than their physical disabilities [6,12]. The psychological toll of tremor may extend beyond the patients themselves. The Clinical Pathological Study of Cognitive Impairment in Essential Tremor (COGNET), a longitudinal study that evaluates cognitive function in older adults with ET, reported that both patients with ET and those close to them suffer psychological stress [13]. In addition, patients may develop feelings of social isolation [11,14] and depression [6,11,13]. Due to the incredible burden put on individuals diagnosed with ET or PD, a multitude of approaches have been investigated to improve the symptoms and quality of life of those afflicted. These range from lifestyle interventions, pharmacotherapy, and surgical treatments.

Lifestyle interventions focusing on the use of weighted utensils can reduce the amplitude of tremor and alleviate the challenges patients face in their activities of daily living (ADLs) [15,16]. With additional weights, these utensils (e.g., spoon) can assist patients to eat and drink. In 2017, the National Institute for Health and Care Excellence (NICE) produced guidelines for the management of PD in adults [5]. Patients in the early stages of PD may benefit from physio- and occupational therapy if they experience motor symptoms or have difficulties with ADLs [5]. However, lifestyle and the nonpharmacological management of ET were not discussed in the guidelines produced by the American Academy of Neurology (AAN) and the IPMDS [17,18,19]. A systematic review of 19 studies found that physical therapy, limb cooling, vibration therapy, use of limb weights, bright light therapy, and transcranial magnetic stimulation were all examples of investigated treatments of tremor [20]. However, these studies mainly included convenience samples, and the long-term effectiveness of these interventions was not assessed [20].

Pharmacotherapy for the treatment of ET is suboptimal and only treats the symptoms. Many patients do not respond to the existing medications indicated for ET and do not experience a significant improvement in their daily living. Currently, propranolol and primidone are the two first-line therapies [15,16,17,18,19,21]. Across randomized controlled trials (RCTs), propranolol and primidone monotherapy produce a mean reduction in the tremor amplitude of 54.1% and 59.9%, respectively, as measured by accelerometry [22]. Nonetheless, 56.3% of patients eventually discontinued the use of either medications [23]. Topiramate is also recommended as a first-line therapy by the guidelines of the Italian Movement Disorders Association (IMDA) [24] and is considered clinically useful at higher doses by the IPMDS task force [19]. However, it is recommended by the AAN guidelines as a second-line therapy [17,18]. Second-line medications have been reported to be less efficacious in reducing the amplitude of tremors. These include alprazolam, atenolol, gabapentin, and sotalol, as well as the aforementioned topiramate [17,18]. In contrast, there is no consensus in the management of PD tremors. The current NICE guidelines recommend levodopa as the first-line therapy for management of all motor symptoms in patients in the early stages of PD [5].

Deep brain stimulation (DBS), whose efficacy has been demonstrated through closed loop approaches [25,26] and interleaving stimulation [27], is the most common surgical treatment to date, providing durable tremor control, especially for patients with medically refractory ET or advanced PD. The effectiveness of DBS in ET and PD tremor is thought to be due to the direct electrical stimulation to the ventral intermediate nucleus (VIM) possibly disrupting the synchronous firing of thalamic neurons [28,29]. In addition to the VIM, the subthalamic nucleus, internal globus pallidus, and pedunculopontine nucleus are also effective targets for DBS in patients with PD tremors [30]. The use of DBS was approved by the Food and Drug Administration (FDA) for ET in 1997, for advanced PD in 2002, and for mid-stage PD in 2016. As of late, radiofrequency thalamotomy has become less favored. An RCT comparing DBS with thalamotomy in 68 patients with tremor due to ET, PD, or multiple sclerosis found that DBS results in fewer adverse effects (*p* = 0.024) and a greater increase in the Frenchay Activities Index score, which assess 15 ADLs. This suggests a greater improvement in the functional status when compared to thalamotomy [31]. Although surgical treatments for tremors, including DBS, stereotactic radiosurgery (SRS), and magnetic resonance-guided focused ultrasound (MRgFUS), are more efficacious than pharmacotherapy [32], the utilization of these procedures remains low. Limiting factors may include high surgical costs [33,34], access to care [35,36], and patient preference [35]. Other perceived barriers to DBS include practitioner preference [34,37], high resource and labor intensity [34,38], and perceptions of serious surgical risk [34,38,39].

Thus, a growing unmet need for safe and effective tremor control and suppression sets the stage for a range of therapies to bridge this gap between lifestyle modifications, pharmacotherapy, and surgical treatment. Using a variety of noninvasive suppression mechanisms, medical devices fit within this gap to provide effective tremor suppression at a lower risk than surgery. The increasing interest in this area has led to the birth of a new classification of external upper limb tremor stimulators. In 2018, the de novo classification request of Cala ONE (Cala Health, Burlingame, CA, USA) received FDA approval [40].

Herein, we focus on the mechanisms of action, safety and efficacy profiles, and clinical applications of different categories of medical devices that are available clinically or previously investigated for tremor suppression. Furthermore, we highlight the limitations of these devices. Such information may then be translated biomechanically and clinically for potential future advancements of medical device for tremor suppression. 

## 2. Early Innovations

Over the past several decades, a variety of different orthotic and stimulatory approaches has been proposed to target or reverse the abnormal rhythmic activities in the neural pathways of the cerebellum and the thalamus. Beginning 1987, Rosen and colleagues proposed several devices that employed energy dissipation to suppress tremors. The damped joystick is a hand control device designed to facilitate the control of wheelchairs and other applications [41,42,43,44]. This device consists of a sealed chamber filled with viscous fluid and a spherical ball that acts as a damping element to suppress the involuntary movements of the position-sensing actuator. The controlled energy-dissipation orthosis (CEDO) is a wheelchair-mounted device that provides velocity-dependent loading with magnetic particle brakes to a limb coupling cuff [45,46,47]. Similarly, the modulated energy dissipation (MED) manipulator also provides damping via magnetic particle brakes with real-time digital control [48,49].

The success of these early works in showing that velocity-dependent loading can attenuate tremor and involuntary motions led to the development of wearable orthosis with mechanical loading. Other approaches, including electrical stimulation systems and assistive feeding devices, have also been proposed. Most of these are classified as Class I medical devices, meaning that they are registered with the FDA but not subjected to any premarketing review.

## 3. Electrical Stimulation Systems

### 3.1. Median and Radial Nerve Excitation

High frequency transcutaneous electrical nerve stimulation (TENS) has been widely studied and used in the treatment of nociceptive and neuropathic pain [50,51,52,53]. The use of TENS in the treatment of movement disorders, including myoclonic dystonia and ET, was first explored by Toglia and Izzo in 1985 [54]. While the exact mechanism of TENS remains unclear, putative mechanisms focus on its ability to modulate the afferent transmission of sensory information from the periphery to the central nervous system (CNS) [50]. Conventional TENS intends to selectively stimulate the large, myelinated peripheral proprioceptive A-beta (A*β*) sensory fibers [50]. The excitation of the A*β* fibers reduces the transmission of the sensory signals elicited by noxious stimulus, thereby reducing the pain perception [55,56,57,58]. These A*β* fibers carry proprioceptive sensory information into the thalamic circuits that are hypothesized to be involved in tremor generation [59]. Most [54,60,61,62,63], but not all [64], studies suggest that treatment with TENS in patients who have tremors was associated with improved muscle strength and tremor reduction. However, sham-controlled randomized trials are needed to confirm these findings due to potential confounding effects associated with the reason for use.

In 2018, Cala ONE was the first wearable transcutaneous electrical nerve stimulator to be approved by the FDA [40]. The newer version of this device, Cala Trio (Cala Health, USA; previously known as Cala TWO), is currently FDA-registered. The PROspective study for SymPtomatic relief of Essential tremor with Cala Therapy (PROSPECT) pivotal trial for Cala Trio was completed in 2019 [65], but it is still waiting for approval by the FDA. Clinically, Cala Trio is designed to replace Cala ONE for use in the transient, symptomatic relief of hand tremors in adults with ET. This device can be worn for therapy on the left or right wrist.

**Figure 1:**
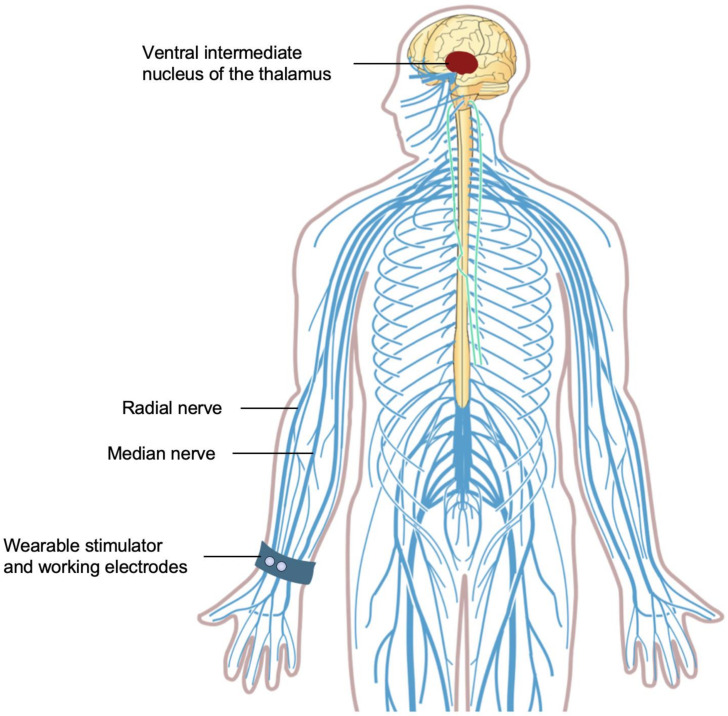
Cala Trio transcutaneous electrical nerve stimulation. The median and radial nerves, which project to the ventral intermediate nucleus of the thalamus, are stimulated by Cala Trio (Cala Health, Burlingame, CA, USA) through two working electrodes placed on the anterior surface of the wrist.

Cala Trio involves two working electrodes positioned over the median and radial nerves on the anterior surface of the wrist and a counter electrode placed on the posterior surface of the wrist. An accelerometer within this device measures the frequency of the patient’s tremor, allowing individualized calibration of the stimulation intensity. The two working electrodes deliver electrical signals that intermittently excite the median and radial nerves in the upper limbs. Peripheral sensory nerves, including the median and radial nerves, also project to the VIM and the neural circuits that are implicated in ET. Similar to DBS, electrical stimulation of the VIM peripherally via the median [66,67] and radial [68] nerves elicits very fast oscillations, which induce thalamicneuronal oscillations and disrupt the pathological oscillations of tremors (Figure 1). A study involving five patients with tremors due to ET or PD demonstrated that electrical stimulation of the median and radial nerves leads to a 57% tremor suppression (*p* < 0.01) [69]. Over time, this stimulation with Cala Trio aims to normalize the neural firing in the pathological tremor network in the CNS to reduce tremors.

The pivotal trial for Cala ONE, a sham-controlled randomized trial of a single 40-min TENS session among 77 patients with ET, found no significant improvements in the Archimedes spiral task, as measured using the Tremor Research Group Essential Tremor Rating Assessment Scale (TETRAS) (*p* = 0.26) [70]. However, the Cala ONE stimulation did show significantly improved upper limb TETRAS tremor scores (*p* = 0.017) and subject-rated Bain and Findley ADL scores (*p* = 0.001), corresponding to a 42% (versus 28% with sham) and a 49% (versus 27% with sham) reduction in tremor amplitude, respectively [70]. The PROSPECT pivotal trial for Cala Trio, an open-label study of TENS treatment in adults with ET, compared twice-daily home therapy TENS sessions over a three-month period among 263 patients [71]. The results, based on 205 patients who completed the study, showed that TENS treatment via Cala Trio resulted in significant improvements in both the TETRAS and subject-rated Bain and Findley ADL scores (*p* < 0.0001) [71]. Among the 193 patients included in the secondary analysis, 54% experienced a ≥50% reduction in tremor amplitude [71]. However, 14 patients did not respond to the therapy, suggesting that not all patients with ET will benefit from Cala Trio [71]. It is important to note that the open label, single-arm design of the PROSPECT trial limits the generality of Cala Trio’s effect; therefore, future studies with more robust designs (e.g., RCTs) would be valuable to assess its efficacy.

Device-related adverse events were mild to moderate in severity. Nonserious adverse events were observed in 18% of patients, including skin irritations (redness, itchiness, and/or swelling); soreness or lesions; and discomfort (stinging and/or sensation of weakness) or burns [71]. These adverse events were all resolved with the use of a topical ointment, decreased stimulation intensity, or discontinued therapy [71]. Contraindications to the use of Cala Trio include having currently implanted electrical medical device (e.g., pacemaker, defibrillator, and deep brain stimulator), suspected or diagnosed epilepsy or other seizure disorders or pregnancy. This device should also not be applied on skin eruptions, open wounds, cancerous lesions, or swollen/infected/inflamed areas.

A 50% reduction of tremor amplitude is comparable to the first-line propranolol and primidone pharmacotherapies [17], which are considered clinically useful for the treatment of ET [19]. Cala Trio can play an important role in patients who are not eligible for surgical intervention or do not respond to pharmacotherapy. It has a similar, favorable safety profile to Cala ONE, whose risk and benefit determination met the FDA’s requirements. This device is noninvasive, with 85% of patients reporting its convenience and ease of use [71]. Currently, it is uncertain whether Cala Trio could reduce or replace the need of medications in the treatment of ET. Thus, physicians need to evaluate how it will fit along with pharmacotherapy and/or lifestyle interventions for each patient with the consideration of tremor severity. Post-approval studies could address this question and provide further insights into the long-term safety and efficacy of Cala Trio.

### 3.2. Antagonistic Muscles Activation

In contrast to TENS, which stimulates sensory nerves, functional electrical stimulation (FES) provides stimulation to motor nerves to trigger muscle contraction. FES for tremor suppression was pioneered by Prochazka and colleagues in 1989 [72,73] and clinically assessed in 1992 [74,75]. Briefly, FES was associated with a tremor suppression of 73% in ET, 62% in PD tremors, and 38% in cerebellar tremors [75]. The recognized limitations of these early works included the potentially unreliable placement of surface electrodes, which could lead to insufficient tremor suppression [74,75]. Although the implantation of percutaneous intramuscular electrodes could solve this problem, this approach is invasive and reserved for patients with severe tremor [64]. Nonetheless, the results from these pilot works led to the first functional electrical stimulator developed specifically for tremor suppression [76,77]. Comparing the previous approach using an analog filter [72,73,74,75], the use of an optimized digital filter [76,77] in a portable functional electrical stimulator, with the enabled self-tuning and adaptation of more complex algorithms, showed improvements in suppressing tremors. In six participants who were healthy or with PD tremor, the functional electrical stimulator based on a digital filter showed an 84% tremor suppression, compared to a 65% when an analog filter was used [77]. The current approaches in utilizing FES to suppress tremors echo these early works, involving primarily two strategies: out-of-phase and co-contraction stimulations [78].

**Figure 2:**
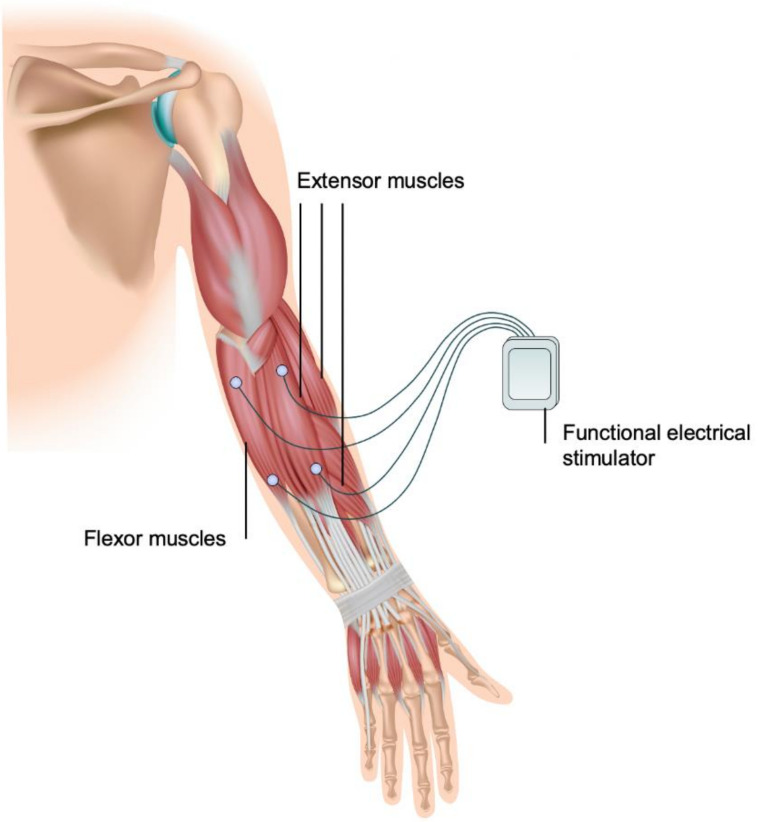
MOTIMOVE functional electrical stimulation system. This device (3F-Fit Fabricando Faber, Serbia) comprises a multichannel stimulator that attaches to several electrodes placed on the flexor and extensor muscles of the forearm, enabling muscle activation.

The MOTIMOVE system (3F-Fit Fabricando Faber, Belgrade, Serbia), based on an out-of-phase stimulation, obtained a CE marking for use in the European Union in 2019 but has not been approved by the FDA (Figure 2). The use of the MOTIMOVE system has been studied in patients with ET, PD tremors [79], and hemiplegia [80]. Two prototypes, the TREMOR neurorobot and the Tremor’s glove, have adapted the co-contraction stimulation. Both devices have been assessed in patients with ET or PD but are currently not approved for clinical use.

The MOTIMOVE system consists of a multichannel stimulator that provides support to activate several electrodes, placed on the forearm and upper arm above the flexor and extensor muscle points, that enable the selective muscle activation via distributed, asynchronous electrical stimulation. The inertial sensors within MOTIMOVE deliver real-time estimation of tremulous movements to a host computer, which provides control over the stimulation of muscles. This system delivers out-of-phase stimulation by sending electrical current pulses to the flexor and extensor muscles, triggering the depolarization of motor neurons that counteracts the tremorgenic activity. A pilot study of MOTIMOVE revealed a 67% tremor suppression in six of seven patients with ET or PD [79]. One patient, however, did not respond, suggesting that out-of-phase stimulation may not work for all patients with tremor [79]. Additional clinical studies evaluating the MOTIMOVE system are claimed to be currently in progress in Serbia, France, and Hungary, which will hopefully demonstrate its efficacy in tremor suppression and feasibility.

The TREMOR neurorobot and the Tremor’s glove adopt a similar design as the MOTIMOVE, consisting of electrodes that provide muscle stimulation, inertial sensors that capture biomechanical characterization signals of tremor, and a controller. Both devices adapt the co-contraction stimulation strategy, which applies mechanical loading via continuous transcutaneous stimulation to a pair of antagonistic muscles, increasing the stiffness of the limb. In turn, this filters out the mechanical manifestation of tremorgenic activity, which are oscillations in the muscle tissue. Like MOTIMOVE, the TREMOR neurorobot stimulates the flexor and extensor muscles of the forearm. This device was found to have a 52% tremor suppression in six patients with ET or PD tremors (*p* < 0.001) [81]. Conversely, the Tremor’s glove stimulates the abductor pollicis brevis and the first and second dorsal interossei muscles of the hand. In a sham-controlled randomized trial of 30 patients with medically refractory tremor in PD, the use of the Tremor’s glove was associated with a significant reduction in the Unified Parkinson’s Disease Rating Scale (UPDRS) score (*p* = 0.001), suggesting improved experiences of daily living and motor complications [82].

The A-alpha (A*α*) sensory fiber, a primary afferent nerve fiber that innervates antagonistic muscle pairs, appears to have a crucial role in the complex neural pathways that are involved in tremor pathophysiology. The reciprocal inhibition of A*α* fibers seems to decrease the excitability of antagonist motor neurons and increase the excitability of agonist motor neurons [83]. While it is not entirely clear whether the reciprocal activation of A*α* fibers results in tremor, intermittent stimulation of the A*α* fibers innervating the flexor and extensor muscles via FES has been studied in patients with ET or PD, showing a 58% tremor suppression [78]. Another study of 14 patients with PD tremor also observed reduction in tremor amplitude and frequency [84]. This suggests that the excitability of antagonist and agonist motor neurons can be modulated, thereby supporting the mechanism by which FES attenuates tremor.

Muscle fatigue is commonly seen as a nonserious adverse event in patients who are treated with FES, owing to the fact that both out-of-phase and co-contraction stimulations lead to the activation of joints and muscle contraction [85]. The use of the Tremor’s glove can also result in numbness of the hand and burning sensation [82]. Contraindications for FES include a prior implanted electrical device, cancer, osteomyelitis, thrombosis/hemorrhage, epilepsy, or pregnancy [86]. In each case, it is incumbent on physicians to evaluate the risk and benefit of a FES treatment based on the patient’s medical history.

Functional electrical stimulators are minimally invasive and demonstrate sufficient efficacy in the suppression of tremor. However, muscle fatigue during repeated FES-induced contraction limits their long-term use. To address this limitation, several emerging technologies have been proposed to reduce or counter muscle fatigue during FES [85]. These functional electrical stimulators have only been studied in small cohorts of patients. Large scale, sham-controlled randomized trials are necessary to validate the efficacy and safety of these devices.

## 4. Wearable Orthoses

The first reported mechanical solution for the suppression of hand tremors was focused on clasping the patient’s arm to prevent involuntary spasms, patented by Terry and Hoyt in 1980 [87]. However, this approach was not developed further. In 1998, the Viscous Beam orthosis [88] was developed based on previously established principles of energy dissipation [45,46,48] to suppress tremor along the wrist flexion/extension. This device showed success, demonstrating that energy dissipation could be employed in an orthosis. However, it was limited by the fixed damping rate, leading to inconsistent tremor suppression [88].

Tremor suppression orthoses for the upper limbs, wrist, and elbow joints are classified into active, semi-active, or passive. Active orthoses work by generating an active force that counteracts the involuntary motions while supporting the voluntary motions in patients with tremors. In contrast, semi-active and passive orthoses leverage energy dissipation or absorption to suppress involuntary movements. Unlike passive orthoses, the damping magnitude of semi-active orthoses can be adjusted by an active controller. Tremelo (Five Microns, Fresno, CA, USA), Steadi-One (Steadiwear, Toronto, ON, Canada), and Readi-Steadi (Readi-Steadi, Gonzales, LA, USA) are the three passive orthoses currently available for use in patients with tremor. Both Tremelo and Steadi-One are FDA-registered, while Readi-Steadi is FDA-exempted. Active and semi-active orthoses are currently being researched but are not clinically available.

### 4.1. Active Suppression

In 2005, the Wearable Orthosis for Tremor Assessment and Suppression (WOTAS) exoskeleton was developed as part of the Dynamically Responsive Interventions for Tremor Suppression (DRIFTS) project of the European Commission [89,90,91,92]. WOTAS consists of sensors that measure rotational motions around the joints, electrical direct current (DC) motors that act as actuators to exert force to suppress tremor by converting electrical energy into mechanical energy, and a controller. This device is placed parallel to the upper limb, suppressing tremor in the wrist flexion/extension and pronation/supination and the elbow flexion/extension. In ten patients with tremors, WOTAS demonstrated a 40% tremor suppression [92]. The major drawback of this device is that it is large and bulky, posing social exclusion concerns [91].

Subsequent active orthoses were developed with similar designs and mechatronics to WOTAS but vary in the types of actuators to reduce the weight and improve the tremor suppression efficacy. The pneumatic actuator, which has a large power-to-weight ratio, was implemented in an orthosis, along with an adaptive tremor estimation algorithm, to suppress tremors in the wrist flexion/extension and adduction/abduction [93,94,95]. The results at the testbench using datasets from ten patients with ET or PD tremor showed a 98.1% tremor suppression [95]. The adaptive disturbance rejection controller, utilizing a permanent magnet linear motor (PMLM), demonstrated a 97.6% tremor suppression when examined with five tremulous signals from patients with PD [96]. Compared to the pneumatic actuator, the PMLM is simpler and faster to control and requires only one sensor [96]. The voluntary-driven elbow orthosis, using an electronically communicated (EC) motor, provided a 99.8% tremor suppression in lab simulation using data from a patient with ET [97]. The wearable tremor suppression glove (WTSG), consisting of an actuation box that includes a multi-channel mechatronic splitter (MMS), aims to provide power support from a single input source to multiple output applications [98]. The MMS incorporates a power EC motor and a steering EC motor to suppress tremors in the wrists and hands [98]. The efficacy of this actuation system, however, has not been evaluated.

Other active orthoses have sought to integrate complex sensor systems to characterize voluntary motions and detect tremors. The myoelectric-controlled upper limb orthosis incorporated an algorithm that recognizes and extracts voluntary movements from surface myoelectric signals [99,100,101,102]. The results from six participants who were healthy or with ET showed a recognition rate of 82% [102]. The newer version of this orthosis adopted a different design that improved flexibility and ease of wear [103,104]. In a healthy participant with FES-induced muscle contraction, the myoelectric-controlled orthosis reduced oscillations in the elbow flexion/extension by about 50–80% [104]. Huen and colleagues implemented context aware body sensor network (BSN) sensors into an upper limb orthosis to enable the detection of six ADLs [105]. In six healthy participants with simulated tremor movements, the BSN-integrated exoskeleton exhibited a 70% accuracy rate in identifying ADLs and a 77% tremor suppression [105].

### 4.2. Semi-Active Suppression

Several semi-active orthoses utilize magnetorheological (MR) fluids as a strategy to provide tremor suppression. MR fluids consist of magnetizable, microscopic particles dispersed in oil or water. Upon encountering a magnetic field, these particles experience attractive force, and the viscosity of the MR fluids increases, opposing the existing flow. This rheological property has been exploited in tremor suppression orthoses by varying magnetic field intensities to tune the resistance force for tremor suppression [106].

The Double Viscous Beam (DVB) orthosis, positioned on the dorsal surface of the forearm, consists of a chamber of MR fluids and two shear plates to make up a passive actuator, applying mechanical loads for tremor suppression [107]. Compared to the previous approach [88], the DVB orthosis has an improved responsiveness to the viscous resistance as a result of the increased shear strength. This orthosis is coupled to a sensor and a controller to optimize the actuation performance.

Case and colleagues developed a wearable orthosis that incorporated four MR dampers for the wrist flexion/extension and abduction/adduction, the elbow flexion/extension, and the forearm pronation/supination [108,109,110,111]. An estimation algorithm for tremor frequency and a controller were used to measure the amount of resistance force needed to counteract tremulous movements. The resistance force generated by the MR dampers depends on a piston-coil design.

More recently, the MR damper-based soft exoskeleton for the tremor suppression (SETS) system was proposed to suppress tremor in the wrist [112]. Unlike previously designed semi-active orthoses, the SETS system equips a controllable flexible semi-active actuator that dynamically adapts to the motions of the wrist joint, providing tremor suppression in the wrist flexion/extension, abduction/adduction, rotation. This device also integrates passive suppression with two hyper-elastic blades, which suppress tremor in the wrist supination/pronation. The SETS system demonstrates potential clinical utility with its compatibility with the human wrist, real-time tunability based on tremor frequency, and lightweight design.

The carbon fiber-based, lightweight orthosis developed by Herrnstadt and Menon is magnetically activated by an electromagnetic brake (EB) [113]. When the tremor frequency and joint angular displacement are detected by a sensor and potentiometer, respectively, a pulse width modulation signal is sent from the controller to produce a magnetic field, exciting the EB. In turn, the EB actuates the orthosis to generate a resistive force for tremor suppression. In comparison to electric motors such as the DC motor, EBs are capable of producing a higher force while consuming less power. In three healthy participants with simulated tremor motions, the use of the EB-based orthosis demonstrated an 88% tremor suppression [113].

Apart from magnetically driven semi-active orthoses, Kalaiarasi and Kumar designed a pneumatically controlled hand cuff [114]. Similarly, this device is built along with an accelerometer that sends tremor frequency data to a controller. When the threshold is met, an air pump inflates the hand cuff, yielding a resistance force in a reciprocating, linear motion to suppress the tremor. Inflation and deflation of the hand cuff are enabled by two separate valves. The limited efficacy of this approach was observed in one patient with ET who experienced a 30% tremor suppression [114].

### 4.3. Passive Suppression

**Figure 3:**
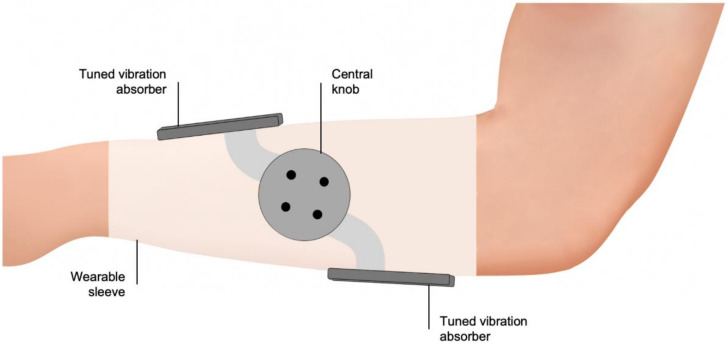
Tremelo passive orthosis. The vibration energy of tremor is being transferred to a mass-spring-damper system within the two tuned vibration absorbers of Tremelo (Five Microns, Fresno, CA, USA), positioned over the dorsal and ventral surfaces of the arm to counteract the involuntary motions.

Tremelo utilized two tuned vibration absorbers (TVAs) that are positioned over the dorsal and ventral surfaces of the arm (Figure 3). Each TVA contains a mass-spring-damper system in which the vibration energy of involuntary motions of the shaking arm during tremor are transferred from the spring to the added mass. This results in reduced tremulous movements and substantial motions of the added mass within the TVA. This device is purely mechanical, eliminating the need of a power source. Preliminary results showed an 85% tremor suppression in a patient with PD tremors [115]. Recruitment for a pilot clinical study is ongoing, which should provide further data.

**Figure 4:**
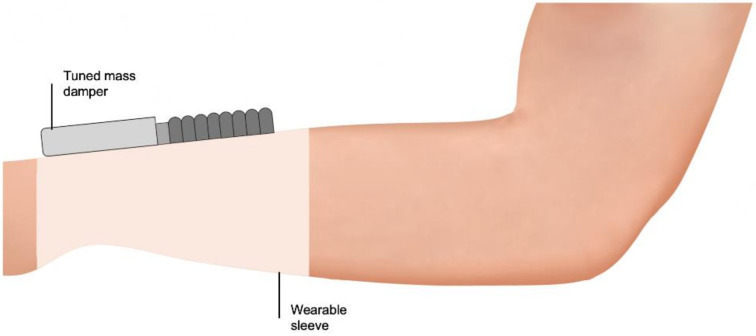
Steadi-One passive orthosis. The non-Newtonian fluid within the tuned mass damper of Steadi-One (Steadiwear, Toronto, ON, Canada) becomes viscous when the vibration energy of tremor is transferred to a mass-spring-damper system, acting as a dissipating element that reduces the amplitude of motions.

Steadi-One is mechanical device that integrates a tuned mass damper (TMD), which obviates the need for a power source [116] (Figure 4). Like TVAs, the TMD embodies a mass-spring-damper system. The difference between TMDs and TVAs is the presence of a dissipating element, which, in Steadi-One, is a non-Newtonian fluid in the interior space of the TMD. When the vibration energy is transferred to the added mass, this non-Newtonian fluid becomes viscous, reducing its amplitude of motions. There are no publicly available data to support its efficacy. However, it is claimed (https://www.steadiwear.com, accessed on 26 March 2021) to have an 85–90% tremor suppression during the lab simulation.

The Readi-Steadi glove embeds a multitude of metal disks that aims to add inertia to the tremulous hand. A preliminary study involving 40 participants who were healthy or with ET observed a 50% tremor suppression [117]. The metal disks function as sensory tricks that influence the aberrant sensorimotor integration to suppress tremor. While there is no study that examines the effectiveness of the sensory trick phenomenon in patients with ET or PD tremor, it has been studied in 30 patients with musician’s dystonia [118]. By wearing a glove, patients with more severe symptoms of dystonia showed better improvements in fine motor control (Pearson’s r = −0.45; *p* = 0.01) [118]. 

The Task-Adjustable Passive Orthosis (TAPO) has a textile glove design to enhance wearability and comfort for daily activities. An air-filled structure, inflated on-demand by hand or electrical pump, is fitted within the glove on the dorsal surface of the hand. The inflated TAPO applies pressure to the back of the hand and the forearm, suppressing the involuntary motions in the wrist flexion/extension, ulnar/radial deviation, and pronation/supination. The proof of concept of TAPO has been examined in a patient with PD tremors performing six ADLs. The use of TAPO was associated with a significant tremor suppression in three specific tasks, including 82% while drinking (*p* = 0.03), 79% while pouring (*p* = 0.03), and 74% while drawing a spiral (*p* = 0.03) [119].

More recently, Lu and Huang examined and established a mechanical model for particle damping for passive vibration suppression in tremors [120]. Particle dampers involve the potential of energy absorption and dissipation through momentum exchange between moving particles and vibrating walls. There are several advantages of using particle dampers, including simple construction, low cost, robustness and reliability, wide damping frequency band, and insensitivity to extreme temperature [120]. At a high tremor frequency, the provided damping of the particle damper became nearly independent to the frequency and amplitude of tremor, indicating that it is suitable for tremor suppression [120].

Two other passive orthoses have also been previously investigated for their use in tremor suppression. The Vib-Bracelet, also designed with an incorporated TMD, suppresses tremors in the wrist pronation/supination [121,122]. The result at the testbench using tremor data from one patient with PD showed an 85% tremor suppression [122]. Another approach, proposed by Takanokura and colleagues [123], involved implementing air dashpots into an orthosis to suppress tremors in the wrist flexion/extension and ulnar/radial deviation, as well as the elbow flexion/extension. In a healthy participant with electrical stimulation-induced muscle contraction, this orthosis demonstrated an involuntary movement suppression of 62% in the wrist when two air dashpots were used and 82% in the elbow [123].

### 4.4. Mechanism Underpinning the Efficacy of Wearable Orthoses in Tremor Suppression

Although some of the underlying causes of tremors remain unknown, several putative interactive factors contributing to the motor expression of tremor have been hypothesized. These include the oscillating tendencies of the joint and muscular mechanical systems, short- and long-loop reflexes of the spinal cord and the brainstem, and the closed-loop feedback systems of higher motor centers such as the cerebellum [124]. Unlike electrical stimulation systems, wearable orthoses target the clinical manifestations of tremors. By generating an opposite force of equal magnitude, these devices attempt to mechanically counteract the involuntary movements. 

### 4.5. Comparing Active, Semi-Active, and Passive Orthoses for Tremor Suppression

Unlike semi-active and passive orthoses, active orthoses often rely on actuators coupled to a signal transmission system, resulting in their heavy and unwieldy nature. ^A^ reduction in the overall weights to improve their wearability is an important research priority. For example, Kelley and Kauffman recently proposed substituting the traditional actuators with the soft and compliant dielectric elastomer stack actuators to enable an orthosis conforming to the human joints [125,126]. In lieu of the metallic structure of previous orthoses, the BSN-integrated exoskeleton leveraged plastic materials to reduce the weight [105]. By using an MMS to support multiple output applications with one drive motor, the WTSG has a reduced size and weight [98].

Wearable orthoses are primarily noninvasive in suppressing tremors. However, their safety profile has not been established, because most wearable orthoses were only assessed in small cohorts of patients or at the testbench with data simulation. Furthermore, the small sample size of these studies may undermine the reliability of the data. It is likely that wearable orthoses will become the most widely used medical devices for tremor suppression, given their promising efficacy. Major challenges include developing orthoses that are lightweight and soft in texture, studying the orthotic placement that will result in maximized tremor suppression, and improving the ergonomic design based on the anatomy of the upper limbs. Addressing these questions with further studies should enhance our understanding of the feasibility and practicality of clinically implementing wearable orthoses to suppress tremor.

## 5. Assistive Feeding Devices

The Neater Eater (Neater Solutions, Derbyshire, UK) was introduced by Michaelis in 1988 [127]. However, it was not available for use in the US until its registration with the FDA in 1993. This is a table-mounted device that involves internal spring-assisted lifting to support a clip-on utensil to enable eating. It relies on viscous damping to absorb tremors and fast movements via the flowing of a viscous fluid that dissipates the kinetic energy. A brief report interviewing 39 participants with various neuromuscular conditions found that the Neater Eater is associated with positive impacts in independence, self-confidence, and quality of life [128]. Follow-up and monitoring may be necessary to prevent fatigue and muscle build-up from using this device on a regular basis. 

Liftware Steady (Verily Life Sciences, South San Francisco, CA, USA), registered with the FDA in 2013, is a handheld device designed to help patients with ET or PD tremors eat. It consists of a motion-generating platform, capable of directing two DC motors to move the utensil opposite to the direction of the tremor. The patient’s involuntary movements are detected by an accelerometer, which are then transmitted to a controller, providing control over the motion-generating platform. A pilot study involving 15 patients with ET demonstrated an improvement in tremor with the device, as measured by Fahn-Tolosa-Marin Tremor Rating Scale (TRS), while holding (*p* = 0.016), eating (*p* = 0.001), and transferring objects (*p* = 0.001) [129]. When using the device, patients expressed improved symptoms of ET while eating (*p* < 0.001) and transferring objects (*p* = 0.013) but not holding them (*p* = 0.14), as measured by the subject-rated Clinical Global Impression Scale (CGI-S) [129]. Data demonstrated a 73% tremor suppression across the three tasks [129]. Compared to other adaptive utensils in 22 patients with ET or PD tremor, Liftware Steady was preferred [130]. 

The Gyenno Spoon (GYENNO Technologies, Shenzhen, China), another handheld assistive feeding device, consists of a longitudinal motor and a transverse motor that are able to generate movements in two different directions opposite to the tremor direction [131]. Both motors are linked to a control module, which receives vibration data from multiple movement sensors within the device. Its ergonomic design and proposed efficacy led to its registration with the FDA in 2016 for use for patients with ET or PD tremors. While it claims (https://www.gyenno.com/spoon-en, accessed on 26 March 2021) to have an 85% tremor suppression, no clinical data has been published.

Contrary to Liftware Steady and the Gyenno Spoon, the Neater Eater requires no power supply and has the ability to automatically bring the spoon forward to the patient’s mouth with an internal spring. However, both Liftware Steady and the Gyenno Spoon enable data collection of the patient’s tremor. This can be beneficial in allowing physicians to monitor their patients’ tremor improvement or progression when Liftware Steady or the Gyenno Spoon is supplemented with pharmacotherapy. Nonetheless, it is important to note that the functionality of these devices is limited to provide support in feeding only.

## 6. Other Devices

### 6.1. Gyroscopic Stabilization

The GyroGlove (GyroGear, London, UK) is developed with a plurality of gyroscopes, mounted to a fabric glove on the dorsal surface of the hand, that function to counteract tremors [132]. Each gyroscope includes a rotatable disc that is capable of rotating about an axis to resist involuntary motions. This allows its angular momentum to be conserved when a rotational displacement is encountered, with opposing force inputs from any direction. Unlike previous gyroscopic devices using a single gyroscope [133,134], the use of multiple gyroscopes in the GyroGlove allows it to suppress involuntary movements in multiple planar directions. This device is currently in the advanced stage of development, and data is needed to inform its efficacy.

### 6.2. Haptic Stimulation Systems

The Emma Watch (Microsoft, Redmond, WA, USA) is a wrist-worn device consisting of several vibration-generating actuators aimed at providing haptic stimulation to the wrist. Mechanosensitive receptors, such as the Pacinian and Meissner corpuscles in the upper limbs, deliver afferent signals to the cuneate nucleus in response to vibratory stimuli [135,136]. Proprioceptive inputs from the cuneate nucleus are projected to the thalamus [136,137,138], implicating its possible role in the neural pathway associated with tremors [139]. However, a study involving 18 patients with ET reported that a mechanical vibration to the hand and forearm via piezoelectric actuators resulted in no homogenous effect in the tremor amplitude [139]. Across different frequencies of vibratory stimuli, 50–72% of patients experienced an increase in tremor amplitude, while 5–22% of patients showed a decrease [139]. The Emma Watch will require clinical validations, since the use of haptic stimulation for tremor suppression has, to date, only led to questionable efficacy.

## 7. Tremor Suppression Devices: Place in Therapy

The onset of ET can occur early in childhood due to familial factors, but the majority of cases of ET appeared after the age of 40 [140]. One study investigated the correlation between the age of onset and the progression of ET in 115 patients [141]. Patients with an age of onset later than 60 years experienced a more rapid progression when compared to patients with a younger age of onset (*p* < 0.001) [141]. Since the onset of ET and PD tremors typically occurs in middle to late adulthood, aging-associated diseases such as dementia [142,143] and mild cognitive impairment [144,145,146] intersect with both of these conditions. These neurological disorders may further preclude patients from adhering to pharmacotherapies.

The medical devices described above offer alternative options for the suppression of tremors (Table 1), especially in patients who are not eligible for surgical interventions (i.e., DBS, SRS, and MRgFUS). However, the use of these devices is patient specific. For example, although Cala Trio has an aesthetic design that will likely not pose any social concerns, wearable orthoses may be a better option if the patient has any contraindication to the use of electrical stimulation systems. Depending on the patient’s needs, assistive feeding devices may be a useful addition to the patient’s daily living. Most of the devices that are available for use are subjected to the FDA’s Class I general control for safety and efficacy assurance. In addition to the general control, Cala ONE requires Class II special control for its performance standards and special prescriber labeling.

ET is associated with a staggering cost of direct medical expenses, indirect productivity and income losses, nonmedical expenses, and disability benefits. The unemployment rate increases to about 88% in patients whose ET progresses from mild to severe [147], leading to forced early retirements. Collectively, patients with mild ET have a 1.83-year average loss of employment, corresponding to a $280 billion in income loss [147]. In patients with moderate to severe tremors, the average loss of employment is 6.5 years [147]. ET and PD tremors likely increase the economic burden more than currently estimated due to their progressive natures and the underreported cases. The development of a medical device for tremor suppression is an under-researched area. Most of the investigational devices discussed were abandoned before entering the market. However, it is imperative that the search for safe and effective tremor suppression devices continues, given the overall economic burden of tremors. Given that most of the currently available devices are based on preliminary data, more investigation is needed to understand the safety and efficacy of these devices before their use in clinical practice can be supported. Cost-effectiveness data are necessary and important to convince insurance programs to provide coverage, alleviating the financial constraints on patients and caregivers.

## 8. Future Perspectives

The devices currently studied have employed distinctive mechanistic approaches. The weight of evidence supporting their efficacy challenges the notion that tremors originate from a single, dominant pathway. Additional pathological insights, such as the loss of Purkinje cells in ET [148,149] and increased central oscillator synchronization in the basal ganglia in PD tremors [150], along with several mechanistic targets of tremor suppression devices, highlight the advances in our understanding of how tremors may be generated. Perhaps the most pertinent pathway implicated in tremors is the cortico-ponto-cerebello-thalamo-cortical loop, which serves as the basis for successful surgical interventions [21]. These findings suggest an integrative multi-pathway model for tremor pathogenesis. The relevance of these pathways necessitates a further clarification of the complexities and inter-related causes of tremors, which is central to spur the future development of safer and more effective devices for tremor suppression.

The lack of consensus on the characterization and electrophysiology of tremor previously represented two major diagnostic pitfalls [151]. However, in 2018, the IPMDS task force reviewed the vast uncertainties to update its consensus classification criteria for tremor disorders [1]. Besides ET and PD tremors, it is important to recognize that a wide range of other tremor conditions also affect the upper limbs with varying clinical features and etiologies [1]. Future studies could investigate whether the efficacy of these devices is generalizable to other tremor conditions. As seen in the pivotal Cala ONE trial [70], tremor suppression can, in part, be attributed to the surgical placebo effect. Since the studies of most of these devices were descriptive in design, sham-controlled randomized trials are warranted to confirm their efficacy. Lastly, evaluating the concurrent use of one or more devices, along with pharmacotherapy/lifestyle interventions, may derive insightful data to explain the benefits and overall impact of a multimodal strategy in the management of tremors.

## 9. Conclusions

Although tremors are not a life-threatening movement disorder, they can be disabling and negatively impact the patient’s quality of life. Our limited knowledge of the pathophysiology of tremors has given rise to the challenge of developing effective or curative pharmacotherapies. In the past several decades, many medical devices, with a broad range of mechanisms, have been developed to suppress tremors in different aspects of daily living. Based on the current evidence, some of these devices appear to have promise as potentially safe and effective options in the medical armamentarium for tremor suppression. Most of these devices are noninvasive and placed externally around the wrists or the upper limbs. It is important to note that externally wearing a device could pose a cosmetic and social concern, so understanding the acceptability of tremor medical devices among patients with tremors is warranted. Nonetheless, it is likely that the future of tremor management will benefit from the addition of medical devices into the patient’s existing pharmacotherapy and/or lifestyle intervention. However, given the high variability in the quality of the current studies, future research is needed to better understand the long-term efficacy, safety, and cost-effectiveness of the tremor suppression devices to fulfill this promise.

## Figures and Tables

**Table 1:** Summary of the tremor suppression devices and study results.

Type of Device	Study Participants (*n*)	Efficacy	Risks	Refs
Electrical Stimulation Systems: Transcutaneous Electrical Nerve Stimulators				
Cala ONE ^‡^	ET (77)	Improved upper limb TETRAS tremor scores (*p* = 0.017) Improved subject-rated BF-ADL scores (*p* = 0.001)	Skin irritations (redness, itchiness, and swelling) Soreness or lesions Discomfort (stinging and sensation of weakness) or burns	[70]
Cala Trio *	ET (205)	Improved upper limb TETRAS tremor scores (*p* < 0.0001) Improved subject-rated BF-ADL scores (*p <* 0.0001)		[71]
**Electrical Stimulation Systems: Functional Electrical Stimulators**				
MOTIMOVE	ET (3); PD tremor (4)	67% tremor suppression	Muscle fatigue	[79]
TREMOR neurorobot	ET (4); PD tremor (2)	52% tremor suppression		[81]
Tremor’s glove	PD tremor (30)	Reduced UPDRS score (*p* = 0.001)		[82]
**Wearable Orthoses: Active Orthoses**				
WOTAS exoskeleton	ET (7); MS tremor (1); Posttraumatic tremor (1); Mixed tremor (1)	40% tremor suppression [92]	Not reported	[89,90,91,92]
Pneumatic actuator-based orthosis	ET (5) ^§^; PD tremor (5) ^§^	98.1% tremor suppression [95]		[93,94,95]
PMLM-based orthosis	PD tremor (5) ^§^	97.6% tremor suppression		[96]
Voluntary-driven elbow orthosis	ET (1) ^§^	99.8% tremor suppression		[97]
MMS-based WTSG	Not reported	Not reported		[98]
Myoelectric-controlled orthosis	ET (2); Healthy (4)	Not reported		[99,100,101,102]
Myoelectric-controlled orthosis (ver. 2)	Healthy (1)	50–80% tremor suppression [104]		[103,104]
BSN-based orthosis	Healthy (6) ^§^	77% tremor suppression		[105]
**Wearable Orthoses: Semi-Active Orthoses**				
Double viscous beam orthosis	Not reported	Not reported	Not reported	[107]
MR damper-based orthosis	Not reported	Not reported		[108,109,110,111]
SETS system	Not reported	Not reported		[112]
Electromagnetic brake-based orthosis	Healthy (3) ^§^	88% tremor suppression		[113]
Pneumatic hand cuff	ET (1)	30% tremor suppression		[114]
**Wearable Orthoses: Passive Orthoses**				
Tremelo *	PD tremor (1)	85% tremor suppression	Not reported	[115]
Steadi-One *	Lab simulation	85–90% tremor suppression		[116]
Readi-Steadi *	ET (20); Healthy (40)	50% tremor suppression		[117]
Task-Adjustable Passive Orthosis	PD tremor (1)	82% tremor suppression while drinking (*p* = 0.03) 79% tremor suppression while pouring (*p* = 0.03) 74% tremor suppression while drawing a spiral (*p* = 0.03)		[119]
Particle Damper	Not reported	Not reported		[120]
Vib-Bracelet	PD tremor (1) ^§^	85% tremor suppression		[121,122]
Air-dashpot-based orthosis	Healthy (1) ^¶^	20–62% tremor suppression in the wrist 82% tremor suppression in the elbow		[123]
**Assistive Feeding Devices**				
Neater Eater *	Not reported	Not reported	Not reported	[127]
Liftware Steady *	ET (15)	Improved FTM-TRS while holding, eating, and transferring objects (*p* = 0.001) 73% tremor suppression		[129]
Gyenno Spoon *	Not reported	85% tremor suppression (claimed)		[131]
**Gyroscopic Stabilizers**				
GyroGlove *	Not reported	Not reported	Not reported	[132]
**Haptic Stimulation Systems**				
Emme Watch	Not reported	Not reported	Not reported	

BF-ADL, Bain and Findley Activities of Daily Living; BSN, body senor network; ET, essential tremor; FTM-TRS, Fahn-Tolosa-Marin Tremor Rating Scale; MMS, multi-channel mechatronic splitter; MS, multiple sclerosis; PMLM, permanent magnet linear motor; SETS, soft exoskeleton for tremor suppression; TETRAS, Tremor Research Group Essential Tremor Rating Assessment Scale; PD, Parkinson’s disease; UPDRS, Unified Parkinson’s Disease Rating Scale; WOTAS, Wearable Orthosis for Tremor Assessment and Suppression; and WTSG, wearable tremor suppression gloves. * FDA-registered; Class I medical device. ^‡^ FDA-approved; Class II medical device. ^§^ Test bench simulation. ^¶^ Induced muscle contraction.

## Data Availability

Data sharing not applicable.
